# Effects of Padel Competition on Brain Health-Related Myokines

**DOI:** 10.3390/ijerph18116042

**Published:** 2021-06-04

**Authors:** Francisco Pradas, María Pía Cádiz, María Teresa Nestares, Inmaculada C. Martínez-Díaz, Luis Carrasco

**Affiliations:** 1 Training, Physical Activity and Sports Performance Research Group (ENFYRED), Faculty of Health and Sports Sciences, Pabellón Polideportivo Río Isuela, E-22001 Huesca, Spain; franprad@unizar.es; 2 Department of Pedagogy, Faculty of Educational Sciences, San Sebastián University, Bellavista 7, Recoleta Piso 6, Santiago 1457, Chile; maria.cadiz@uss.cl; 3 Department of Physiology, Faculty of Pharmacy, University of Granada, Cartuja University Campus, E-18071 Granada, Spain; nestares@ugr.es; 4 Department of Physical Education and Sport, University of Seville, E-41013 Seville, Spain; martinezdiaz@us.es

**Keywords:** padel, sports competition, myokines, BDNF, leukemia inhibitory factor, irisin

## Abstract

Padel is becoming one of the most widespread racket sports that may have potential health benefits. Considering that several myokines mediate the cross-talk between skeletal muscles and the brain, exerting positive effects on brain health status, this study was designed to evaluate the responses of brain-derived neurotrophic factor (BDNF), leukemia inhibitory factor (LIF), and irisin (IR) to padel competition in trained players and to determine whether these responses were sex-dependent. Twenty-four trained padel players (14 women and 10 men with a mean age of 27.8 ± 6.3 years) participated voluntarily in this study. Circulating levels of BDNF, LIF, and IR were assessed before and after simulated padel competition (real playing time, 27.8 ± 8.49 min; relative intensity, 75.2 ± 7.9% maximum heart rate). Except for BDNF responses observed in female players (increasing from 1531.12 ± 269.09 to 1768.56 ± 410.75 ng/mL), no significant changes in LIF and IR concentrations were reported after padel competition. In addition, no sex-related differences were found. Moreover, significant associations between IR and BDNF were established at both pre- and post-competition. Our results suggest that while competitive padel practice stimulates BDNF response in female players, padel competition failed to boost the release of LIF and IR. Future studies are needed to further explore the role of these exercise-induced myokines in the regulation of brain functions and to identify the field sports that can contribute to myokine-mediated muscle–brain crosstalk.

## 1. Introduction

Padel is a particular racket sport that has grown in popularity and is currently being practiced by millions of people worldwide [1]. Padel is played in doubles on a rectangular court (20 × 10 m) divided into two fields by a central net that is lower than the tennis one. Moreover, this court is completely surrounded by a suitable combination of concrete walls and fence wire, which prevent the ball from exiting the playing area [2,3].

As in other racket sports, several studies have been focused on the assessment of players’ court-movement patterns and the physiological demands of padel competition. Cardiorespiratory responses (oxygen consumption and mean heart rate) as well as perceived exertion rates are similar to those previously found in tennis. However, they are lower compared to those assessed in squash and badminton [1]. More specifically, and considering that padel practice is characterized by alternated intervals of intense and moderate-low exercise intensity, the mean VO_2_ measured during an official competition (lasting around 1 h) reached values below 50% of maximum VO_2_ (VO_2max_), whereas the mean HR represented approximately 74% of maximum HR (HR_max_) [2]. Thus, moderate energy expenditure (with aerobic metabolism as the main energy source) and an easy and accessible technique seem to be the two key factors behind the extended practice of padel.

However, although padel practice keeps increasing there remains a lack of information about its impact on players’ health. According to the reviewed scientific literature, there are not many studies dealing with the physiological, health-related effects of high-level padel competition [2]. To our knowledge, only one very recent study has been aimed at analyzing the changes in hematological and biochemical parameters induced by competitive padel practice. As can be observed, high-level padel competition provokes a significant increase in muscle damage biomarkers (e.g., creatine kinase) as well as remarkable decreases in blood electrolytes concentrations [4]. Nevertheless, apart from these intensity-related effects it would be necessary to conduct studies focused on determining the stimulating effects of competitive padel practice on different health-related benefits through specific biomarkers.

Brain-derived neurotrophic factor (BDNF) is an important neurotrophin that plays important roles in the plasticity of several regions of the central nervous system (CNS) during development, adulthood, and aging [5]. Nevertheless, it has been suggested that BDNF is expressed in non-neurogenic tissues (including skeletal muscle), so that BDNF may play a role not only in CNS plasticity but also as a metabolic regulator of skeletal muscle (enhancing glucose consumption and fat oxidation). In any case, of all neurotrophins, BDNF seems to be the most susceptible to regulation by exercise and physical activity [6]. In fact, the response of BDNF to acute exercise has been investigated using different exercise protocols and, consequently, different results have been reported (from a lack of response to increases anywhere between 11.7 and 410.0% with respect to basal levels) [7].

The skeletal muscle acts as a secretory organ that, in addition to BDNF, produces cytokines and other muscle fiber-derived peptides called myokines [8]. In general, myokines are muscle-derived molecules that exert physiological functions on maintaining systemic homeostasis. Thus, myokines regulate whole-body metabolism, bone growth, satellite cell proliferation, and muscle hypertrophy in an autocrine, paracrine, or endocrine manner [9,10]. Irisin (IR) and leukemia inhibitory factor (LIF) are two novel myokines that are associated with brain and muscle adaptations. A recent study has reported that IR is involved in whole-body metabolism regulation by stimulating FFA, oxidation, and lipolysis and inducing fat browning. Moreover, IR stimulates glucose uptake and regulates muscle growth [11]. Although the primary source of IR is skeletal muscle, another source of this myokine is the brain. Therefore, IR could play roles in mediating the effects of physical activity on the brain [12]. On the other hand, LIF is produced by skeletal muscle and affects intact muscles as well as isolated muscle cells. Among its various roles, the most important role of LIF in muscle satellite cell is the proliferation of proper muscle hypertrophy and regeneration [10,13].

However, considering the beneficial effects of BDNF, LIF, and IR on brain health status, there is a lack of information about the magnitude of their responses to field sports activities [14]. As it has been reported in a recent study, the amount of evidence for the effects of exercise on the blood concentration of BDNF is moderate [15]. Moreover, studies on the response of IR to exercise have not been conclusive [16], and although LIF seems to play an autocrine role within the skeletal muscle tissue, it has been difficult to determine changes in their circulating levels in response to exercise.

Thus, taking into account the increasing popularity of padel and the potential brain health effects of BDNF, LIF, and IR, this study was designed to evaluate the responses of these myokines to padel competition in trained players and to determine whether they are sex-dependent.

## 2. Materials and Methods

### 2.1. Participants

A total of 24 trained padel players (14 female and 10 male young-adult players) with more than five years of experience in the professional circuit World Padel Tour participated voluntarily in this study. The characteristics of the subjects can be seen in Table 1. All of the participants gave their informed consent for inclusion prior to participation. The study was conducted in accordance with the Declaration of Helsinki, and the protocol was approved by the Ethics Committee of the Department of Health and Consumption of the Government of Aragon, Spain (code: 21/2012; date: 19 December 2012).

The calculations for sample size and power were based on BDNF responses to moderate and vigorous exercise reported by a previous study [17]. Considering the large effect sizes (ES) shown by these authors (e.g., Cohen’s d range of 0.63–1.16), the a priori sample size calculation (G*Power v.3.1) with ES = 0.64 established that a sample of 22 would be sufficient to obtain a statistical power of 0.8 (*p* < 0.05). Therefore, our sample size of 24 allowed us to overcome a power of 85%.

### 2.2. Experimental Approach

**Figure 1:**
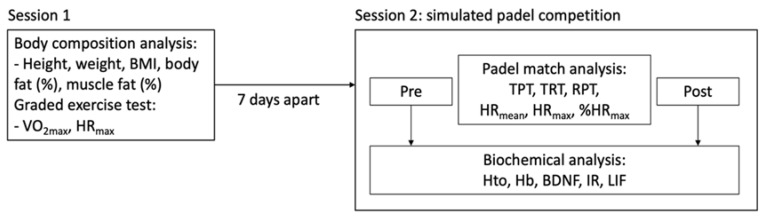
Study protocol.

Participants were involved in two separate testing sessions with at least seven days between them (Figure 1). In the first session, subjects’ body composition analysis was assessed (bioelectrical impedance, TANITA BC–418MA, Amsterdam, The Netherlands) and a graded exercise test was also performed. The second session consisted of participating in a simulated competition following the International Padel Federation rules.

For each session (conducted between 9:00 a.m. and 12:00 a.m.), participants were instructed to avoid strenuous physical activity during the previous 24 h and to abstain from food (overnight fasting), caffeine, and alcohol 12 h before testing. Padel matches were held on outdoor courts with a relative environmental humidity and temperature of 45.7 ± 7.3% and 24.1 ± 7.1 degrees Celsius, respectively.

#### 2.2.1. Session 1: Determination of Cardiorespiratory Fitness

Players’ maximum oxygen consumption (VO_2max_) and maximum heart rate (HR_max_) were assessed in the first session using an incremental running test on a treadmill (Pulsar HP Cosmos, Nussdorf, Germany) equipped with a gas analyzer (Oxycon Pro. Jaegger, Germany) and heart rate monitor (Cosmos, Nussdorf, Germany). After a warm-up period of 5 min of brisk walking (6 km/h), the initial speed was set at 8 km/h, increasing by 1 km/h every minute until volitional exhaustion. The treadmill slope was kept at 1°. VO_2max_ was defined following the ACSM criteria [18], whereas HR_max_ determined as the highest HR value observed during the running test.

#### 2.2.2. Session 2: Simulated Padel Competition Analysis

All matches were played to the best of three sets. If the situation of six equal games was reached, a tie breaker was played. Before each match, players performed a standardized 15 min warm-up. Total playing time (TPT, full time of the match from the beginning to the end, considering the periods of game and rest), total resting time (TRT, sum of periods in which the ball was not in play), and real playing time (RPT, total playing time minus total resting time) were measured (s) for each match. Moreover, players’ HR was continuously recorded during the competition (Polar Team System, Kempele, Finland) as average values over 5 s.

*Blood sampling.* In the second session, two 5-mL blood samples (pre- and post-padel competition) were drawn from the antecubital vein of each participant. Blood samples were collected in Vacutainer tubes (BD Vacutainer, Plymouth, UK) containing ethylenediaminetetraacetic acid (EDTA) as an anticoagulant. The first sample was collected 90 min before the competition (fasting conditions), and the second was collected within 10 min after the matches. Blood samples were immediately put on ice and transferred to a laboratory for processing.

*Hematological and biochemical assessment.* Hematological parameters (red blood cells, hematocrit, and hemoglobin) were determined using the Coulter counter model Ac·T diff (Beckman Coulter Inc., Brea, CA, USA). BDNF concentrations were measured using the BDNF Exam Immunoassay System kit (R&D Corp., Minneapolis, MN, USA) according to the manufacturer’s instructions. Whole blood LIF concentrations were determined by enzyme-linked immunosorbent assay (ELISA) using a human LIF ELISA kit (PromoKine, Heidelberg, Germany) following the manufacturer’s instructions. On the other hand, IR blood levels were measured using a commercial kit (Wuhan Fine Biological Technology Co., Ltd., Wuhan, China). Absorbances for LIF and IR were measured in duplicate using a spectrophotometric microplate reader at a wavelength of 450 nm (Biotek, Winooski, VT, USA).

Lastly, considering that the players were allowed to hydrate ad libitum during the competition (with bottled mineral water), changes in plasma volume were calculated using hematocrit and hemoglobin values following the methods of Dill and Costill [19]. Moreover, blood levels of BDF, LIF, and IR were individually corrected according to the formula described by Matomäki et al. [20].

The data are expressed as mean ± standard deviation (sd). The Shapiro–Wilk test was applied to test for a normal distribution of variables. Parametric and non-parametric tests (ANOVA-time × sex interaction-, Wilcoxon signed-rank, and Mann–Whitney tests) were used where appropriate to determine both intragroup and intergroup differences between the pre- and post-competition time points. Moreover, effect size (ES) was calculated using both partial eta squared (η^2^p) and the d-value proposed by Cohen [21]. Thus, the ES was interpreted as trivial when η^2^p < 0.01 and d < 0.19; small when η^2^p = 0.01 and d = 0.20; medium when η^2^p = 0.06 and d = 0.50; and large when η^2^p = 0.14 and d = 0.80. Bivariate correlations were also performed using both Pearson’s *r* and Spearman’s *rho*, which was set at 0.500 for a positive correlation. For all tests, a *p*-value of <0.05 was used to indicate statistical significance.

## 3. Results

### 3.1. Participants’ Characteristics and Cardiorespiratory Fitness

Body composition variables showed significant sexual dimorphism. Moreover, VO_2max_ was significantly higher in male than in female players (Table 1).

### 3.2. Characteristics of Simulated Padel Competition

As it can be seen in Table 2, one of the most peculiar characteristics of padel competition was the 1:1.5 ratio established between TPT and TRT. Although TPT and TRT showed sex-related differences (both variables were higher in males), no differences were found in RPT.

On the other hand, HR_mean_ and HR_max_ during padel competition were similar in both males and females. Likewise, considering that HR_max_ measured during maximal exercise test did not report sex differences, the percentage of HR_mean_ on reference HR_max_ (graded exercise test) did not show any statistical significance. Regarding PV changes, a slight but not statistically significant increase (+1.1 ± 2.3% for the total group; CI-95%: −0.6 to 3.1) was observed (Table 2).

### 3.3. BDNF, LIF, and IR Responses

**Figure 2:**
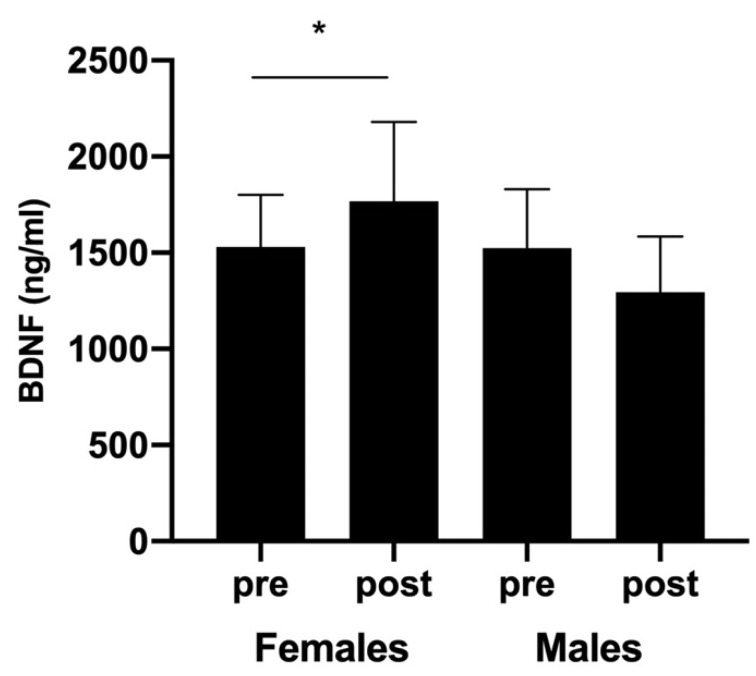
Blood BDNF concentrations (ng/mL) measured before and after padel competition. * *p <* 0.05.

Although no sex-related differences were found, padel competition induced a significant increase in circulating BDNF levels in female players (from 1531.12 ± 269.09 to 1768.56 ± 410.75 ng/mL; CI-95%: −507.20 to 32.32; Z = −2.27, *p* < 0.05, d = 1.527). In contrast, BDNF concentrations measured in males after exercise were lower than those found before (1523.01 ± 307.10 and 1295.51 ± 288.88 ng/mL, respectively). However, no significant differences were observed (CI-95%: −52.61 to 507.61; Z = −0.866, *p* = 0.186, d = 0.476; Figure 2).

### 3.4. Statistical Analysis

**Figure 3:**
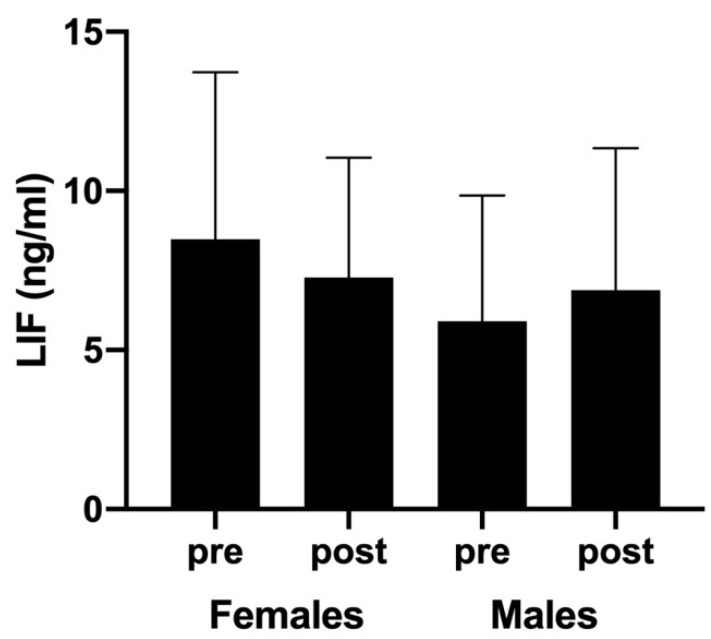
Blood LIF levels (ng/mL) measured before and after padel competition.

Regarding LIF responses, no significant differences were observed when sex (F(3,43) = 0.590; *p* = 0.447, η^2^p = 0.014), time (F(3,43) = 0.004; *p* = 0.952, η^2^p < 0.01), and sex × time interaction (F(3,43) = 0.318; *p* = 0.576, η^2^p < 0.01) analyses were performed. Nevertheless, post-exercise LIF concentrations showed a slight decrease in females (from 8.48 ± 5.25 to 7.28 ± 3.76 ng/mL after competition; CI-95%: −2.35 to 4.75) but a nonsignificant increase in males (from 5.91 ± 3.95 to 6.88 ± 4.46 ng/mL, respectively; CI-95%: −4.93 to 2.99) (Figure 3).

**Figure 4:**
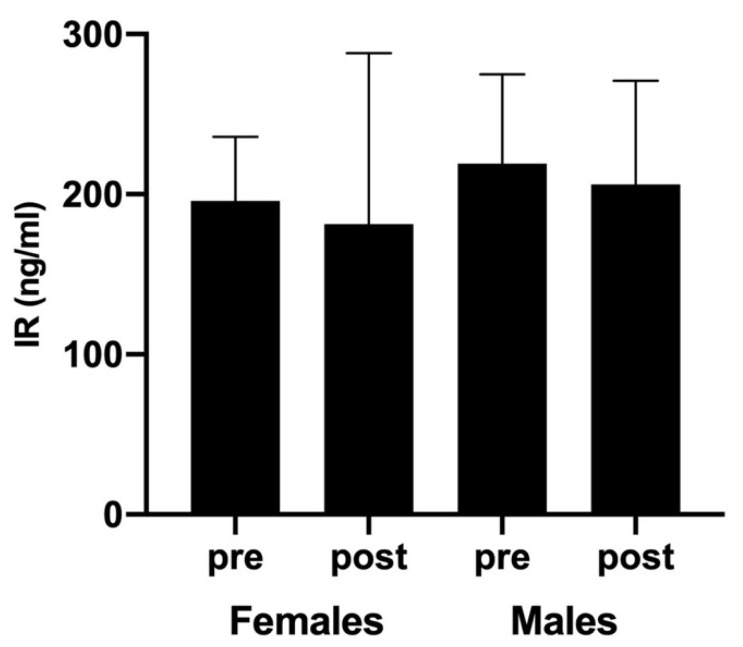
Blood IR concentrations (ng/mL) measured before and after padel competition.

On the other hand, intergroup analysis revealed no significant sex-related differences for IR responses (CI-95%: −158.64 to 111.78, Z= −0.205, *p* = 0.837, d = 0.084 and CI-95%: −142.48 to 92.88, Z = −0.312, *p* = 0.755, d = 0.096 before and after padel competition, respectively). Moreover, unlike BDNF and LIF, the same decreasing trend of IR levels was observed in both female and male players after padel competition. Nevertheless, no significant differences regarding pre-competition levels were found (CI-95%: −89.34 to 118.49, Z = −0.594, *p* = 0.552, d = 0.322 for females, and CI-95%: −142.36 to 168.68, Z = −0.051, *p* = 0.959, d = 0.027 for males; Figure 4).

Finally, a correlation analysis reported significant associations between BDNF and IR for the entire group in both before (rho = 0.461; *p* = 0.024), and after competition (rho = 0.665; *p* < 0.001).

## 4. Discussion

The main aims of this study were to evaluate the responses of BDNF, LIF, and IR to padel competition in trained players and to determine whether these responses were sex-dependent. To our knowledge, there have not been many studies evaluating the responses of neurotrophic factors and specific myokines to competitive sports practice. As has been indicated, padel is increasingly practiced by more people, so it is important to define all of its potential health benefits.

According to previous findings, as was expected, the padel players evaluated in our study showed sexual dimorphism in body composition [22] and cardiorespiratory variables [23]. Moreover, sex-related differences were also observed in padel competition characteristics. TPT and RPT were higher in male than in female players. However, RPT did not show any differences between groups. While these sex-related effects were in accordance with the findings of a very recent study [4], they were contrary to those reported by Torres-Luque et al. [24], who found higher TPT and RPT in female players. In any case, TPT in our study was established approximately between 55 and 80 min, which is in line with these previous reports.

On the other hand, we assessed cardiovascular responses to padel competition using HR_mean_ during matches and its percentage on HR_max_ measured during a graded exercise test. Our results are similar to those previously described by Castillo-Rodriguez et al. [1], who reported a HR_mean_ equivalent to 77% of HR_max_ during padel competition. Only one previous study [4] has considered the impact of padel practice on the hemodynamic variables that could modulate other physiological and/or biochemical responses in padel players. Accordingly, it seems necessary to check PV changes associated with intense outdoor exercise, especially if athletes need to hydrate to replace the water lost through sweating and to maintain an adequate thermoregulation. Thus, taking into account that our padel players kept hydrated during the competition, it was necessary to calculate PV changes to avoid any hemoconcentration or hemodilution that could affect the results. Nevertheless, although a mean increase of 1.1% in PV was measured after padel competition, biochemical data were individually corrected.

Biochemical analysis was focused on BDNF, LIF, and IR, a group of myokines that, among others, could explain the underlying biological mechanisms of neuroprotective and regenerative potential effects of exercise in both CNS and the periphery [25]. In fact, increased levels of multiple myokines that have beneficial endocrine effects play crucial roles in the interactions between skeletal muscle and other organs in response to exercise [11]. Nonetheless, our study showed that, with some exceptions, padel competition fails to induce remarkable changes in circulating levels of these biomarkers. Moreover, it is important to note that their responses were characterized by large interindividual variability.

The response of BDNF to acute exercise has been investigated by several authors using different exercise protocols and, consequently, different results have been reported [7]. In our study, padel competition induced only a slight but significant increase in the circulating BDNF concentrations of female players. This attenuated response is in line with those observed in previous studies in which low or moderate-intensity exercise was used [26,27]. The magnitude of BDNF increase seems to be exercise intensity-dependent, since high-intensity exercises induced huge BDNF responses [7,28], whereas low or moderate-intensity exercises were insufficient to do so [26]. Thus, padel competitions that consisted of 55–80 min of discontinuous exercise (RPT from 22 to 33 min) performed at 72–77% of HR_max_ may fail to stimulate large BDNF responses.

Although LIF is a myokine mainly associated with muscle regeneration, various studies have demonstrated the importance of LIF at various stages of neurogenesis and its involvement in both neuronal cell differentiation and neuritic outgrowth [29]. Previous studies demonstrated that aerobic exercise induces the expression of LIF in human skeletal muscle [13,30]. However, our results reported no changes in LIF circulating levels after padel competition. Moreover, we did not observe differences regarding the players’ sex. Similar results were found by Donnikov et al. [31], who reported a slight decrease in LIF concentrations in a group of athletes after a six hour marathon ultra-race. This lack of LIF responses to exercise could be explained by many different hypotheses. First, as it has been previously observed, exercise-induced LIF responses are characterized by a remarkable interindividual variability. In fact, both increases and decreases in circulating post-exercise LIF levels have been measured in athletes [31]. Second, LIF seems to be muscle-specific, as LIF was undetectable in plasma. It is possible that LIF is secreted to the interstitial space between muscle fibers and does not easily reach circulation. Third, considering the short blood half-life of LIF (6–8 min), it could be difficult to detect accumulated circulating levels of LIF protein during prolonged exercise [32]. Finally, it seems that resistance exercises (mainly eccentric muscle contractions) regulate LIF secretion better than aerobic exercises [13], which would also explain the attenuated LIF responses observed here.

Previous studies have confirmed that IR may stimulate both neuronal proliferation and differentiation [33,34]. Furthermore, IR contributes to the neuroprotective effect of exercise against brain disorders [35]. Although the primary source of IR in humans is skeletal muscle, which produces over 70% of the total circulating level of IR, other non-muscle sources of this myokine (such as the brain) play roles in mediating the effects of physical activity on the brain [12]. In any case, previous results reported that plasma IR levels were elevated in humans following exercise [36]. Thus, it was to be expected that circulating IR levels were increased after padel competition. However, we observed no effect of exercise on IR blood concentrations, since post-exercise values were similar to those measured before padel matches. At the same time, no sex-related differences were detected when both pre- and post-exercise IR levels were contrasted between male and female players. These results are in line with those obtained by Pekkala et al. [37] and Tsuchiya et al. [38], who did not find changes in IR concentrations after 1 h of aerobic cycling at intensities of 50% and 65% VO_2max_, respectively. In this sense, it seems that exercise-related IR response is intensity-dependent, since various studies have demonstrated significant IR acute responses when both trained and untrained subjects performed high-intensity exercises [39,40]. Moreover, after comparing resistance exercise with interval- and endurance-type exercise protocols, Huh et al. [41] reported that, as occurs with LIF, strengthening exercise induced a greater IR response than continuous cardiovascular ones.

Nevertheless, contrary to the findings of Briken et al. [25], correlation analyses revealed significant associations between IR and BDNF in both pre- and post-exercise evaluations. On the basis of these associations, it is important to consider that exercise-related IR response is stimulated by an increased expression of PGC1-α, a regulator of mitochondrial biogenesis. PGC1-α is an inducer of fibronectin type III domain-containing protein 5 (FNDC5) expression, a single-pass membrane-spanning protein. Upon exercise, the ectodomain of FNDC5-IR is released into the bloodstream. Interestingly, FNDC5 was shown to mediate beneficial CNS effects of endurance exercise by upregulating BDNF expression [42]. In any case, future studies should explore this type of relationship and its potential benefits for brain health.

Lastly, as with the majority of studies performed on sports competition, there are some limitations inherent to the design used that should be noted. First, simulated padel competition could be quite different from real padel competition. Second, taking into account the interindividual variability shown by the outcome variables, the sample size was relatively small. However, considering the recruiting difficulties inherent to competitive athletes, the participants in our study were homogeneous in terms of padel competitive category and training status.

## 5. Conclusions

Our results suggest that competitive padel practice induces a slight but significant response of BDNF in female players. However, padel competition failed to stimulate the release of LIF and IR. Padel competition characteristics and relative playing intensity could explain this lack of stimulating effect. In addition, IR and BDNF showed an interesting association that needs to be studied in future research. Nevertheless, with respect to practical applications the findings of our study suggest that padel could be included as a part of programs promoting brain health, especially for women.

## Figures and Tables

**Table 1:** Padel players’ characteristics.

	Females	Males	Total	Sig. (CI = 95%)
Age (years)	29.1 ± 3.8 (26.5–31.6)	26.3 ± 8.2 (20.4–32.2)	27.8 ± 6.3 (24.9–30.6)	*p* = 0.323
Height (cm)	167.1 ± 5.7 (163.3–170.9)	177.1 ± 2.8 (175.1–179.1)	171.9 ± 6.8 (168.8–174.9)	*p* < 0.001
Weight (kg)	60.7 ± 4.5 (57.6–63.7)	76.7 ± 6.2 (72.2–81.1)	68.3 ± 9.7 (63.8–72.7)	*p* < 0.001
BMI (kg/m^2^)	21.7 ± 1.0 (21.0–22.4)	24.4 ± 1.8 (23.1–25.7)	23.0 ± 1.9 (22.1–23.9)	*p* < 0.001
Body fat (%)	20.2 ± 2.1 (18.8–21.7)	13.4 ± 5.1 (9.7–17.0)	16.9 ± 5.1 (14.6–19.3)	*p* = 0.001
Muscle mass (%)	37.1 ± 2.9 (35.1–39.0)	43.3 ± 2.2 (41.7–44.9)	40.1 ± 4.1 (38.2–41.9)	*p* < 0.001
VO_2max_ (mL/kg/min)	47.5 ± 4.9 (44.2–50.8)	57.5 ± 5.7 (53.4–61.6)	52.3 ± 7.3 (48.9–55.6)	*p* < 0.001
HR_max_ (bpm)	186.2 ± 7.8 (181.0–191.5)	188.3 ± 10.7 (180.7–195.3)	187.2 ± 9.1 (183.1–191.4)	*p* = 0.622

CI, confidence interval; BMI, body mass index; VO_2max_, maximum oxygen consumption; HR_max_, maximum heart rate measured in the graded exercise test. Numbers in brackets represent the mean 95% CI of the mean. Italics are used to highlight statistical significance.

**Table 2:** Characteristics of simulated padel competition.

	Females	Males	Total	Sig. (CI = 95%)
TPT (s)	3495.9 ± 1165.1 (2521.9–4469.9)	4760.0 ± 1074.7 (3766.0–5753.9)	4085.8 ± 1264.8 (3385.4–4786.3)	*p* = 0.05
RPT (s)	1490.8 ± 480.7 (1088.9–1892.7)	1872.5 ± 496.1 (1413.7–2331.3)	1668.9 ± 509.8 (1386.6–1951.2)	*p* = 0.155
TRT (s)	1961.7 ± 687.2 (1387.2–2536.3)	2825.7 ± 647.1 (2227.3–3424.1)	2364.9 ± 783.9 (1930.8–2799.0)	*p* = 0.027
HR_mean_ (bpm)	142.4 ± 11.8 (135.9–149.0)	145.4 ± 18.2 (134.3–156.4)	143.8 ± 14.9 (138.0–149.6)	*p* = 0.615
HR_max_ (bpm)	167.8 ± 11.4 (161.4–174.1)	173.8 ± 17.8 (163.1–184.6)	170.6 ± 14.8 (164.9–176.3)	*p* = 0.289
% HR_max_	77.2 ± 5.8 (73.1–81.4)	72.7 ± 9.8 (64.5–81.0)	75.2 ± 7.9 (71.3–79.2)	*p* = 0.245
PV changes (%)	+1.5 ± 2.7 (−0.1–3.1)	+0.5 ± 1.6 (−0.6–1.7)	+1.1 ± 2.3 (−0.6–3.1)	*p* = 0.351

CI, confidence interval; TPT, total playing time; RPT, real playing time; TRT, total resting time; HR_mean_, mean heart rate assessed during padel competition; HR_max_, maximum heart rate assessed during padel competition; %HR_max_, percentage of HR_mean_ on reference HR_max_ (graded exercise test); PV, plasma volume. Numbers in brackets represent the 95% CI of the mean. Italics are used to highlight statistical significance.

## Data Availability

The data presented in this study are available upon request from the corresponding author.
